# Traumatic lumbosacral instability: part 2—indications and techniques for surgical management

**DOI:** 10.1007/s00402-025-05752-9

**Published:** 2025-02-01

**Authors:** Richard A. Lindtner, Dietmar Krappinger, Jan Lindahl, Carlo Bellabarba

**Affiliations:** 1 Department of Orthopaedics and Traumatology, Medical University of Innsbruck, Innsbruck, Austria; 2 Department of Orthopaedics, Harborview Medical Center, University of Washington School of Medicine, Seattle, WA 98104 USA; 3 Department of Orthopaedics and Traumatology, Helsinki University Hospital and University of Helsinki, Helsinki, Finland

**Keywords:** Traumatic lumbosacral instability, Spinopelvic dissociation, Lumbosacral dislocation, Lumbosacral facet joint, Sacral fracture, Treatment

## Abstract

Traumatic lumbosacral instability (TLSI) refers to a traumatic disruption of the axial skeleton at the level of the lumbosacral motion segment and/or sacrum, resulting in mechanical separation of the caudal spinal column from the posterior pelvic ring. Managing TLSI and its four underlying conditions poses unique challenges among spinal and pelvic injuries. This second part of a two-part series focuses on treatment strategies and decision making in TLSI, with an emphasis on surgical stabilization techniques. The primary objectives of this article are to: (1) elucidate factors influencing clinical decision-making, (2) synthesize current treatment options for the injury patterns underlying TLSI, and (3) briefly outline expected outcomes and complications.

## Introduction

Traumatic lumbosacral instability (TLSI) refers to a traumatic disruption of the axial skeleton at the level of the lumbosacral motion segment and/or sacrum, resulting in a mechanical separation of the caudal spinal column from the posterior pelvic ring [1]. TLSI arises from the following four distinct groups of complex bony and/or disco-ligamentous lumbosacral injuries, which form the basis for its classification into four types: lumbosacral dislocation injuries (LSDI; Type 1 TLSI), spinopelvic dissociation injuries (SPDI; Type 2 TLSI), unilateral vertical sacral fractures (UVSF) with ipsilateral lumbosacral facet joint disruption (Type 3 TLSI), and bilateral vertical sacral fractures without a transverse fracture component (BVSF; Type 4 TLSI). While all these injury patterns result in TLSI, they differ in their specific sites of disruption and the resulting fracture fragments.

The management of TLSI and its underlying conditions presents a unique challenge among spinal and pelvic injuries. First, these injuries are complex and may not only affect lumbosacral but also posterior pelvic ring stability. Second, the treating surgeon’s experience with these injuries is typically limited due to their rarity [2–8]. Third, because these injuries occur at the lumbopelvic junction, there is significant anatomical overlap between pelvic surgery and spine surgery. Effective treatment, therefore, necessitates expertise in both pelvic ring fixation and lumbopelvic stabilization techniques [9, 10]. Fourth, there remains considerable debate regarding the optimal treatment strategies, as current treatment recommendations are primarily based on retrospective case series and expert opinions, with a notable lack of prospective studies comparing various treatment approaches [2, 6, 11–13]. Finally, individual injury patterns associated with TLSI have often been examined in isolation rather than within the broader context of TLSI, highlighting the need for a comprehensive synthesis of current treatment concepts.

Therefore, the objectives of this second part of a two-part series are to: (1) elucidate the factors influencing clinical decision-making, (2) synthesize current treatment options for the injury patterns underlying TLSI, and (3) briefly outline the expected outcomes and complications.

## Treatment decision-making

TLSI and its associated injury patterns typically result from high-energy trauma and are frequently accompanied by multiple associated injuries [2, 6, 13, 14]. Initial stabilization and management of these often polytraumatized patients demands a multidisciplinary approach to determine treatment priorities and to effectively address potentially life-threatening conditions. Similarly, definitive surgical treatment of TLSI may require collaborative efforts among specialists. The lumbopelvic junction represents a frontier between the spine and pelvis, as well as between the spine surgeon and the pelvic surgeon. In some trauma centers, the treating surgeon may have extensive expertise in both spinal and pelvic trauma, enabling comprehensive care. However, in other centers, there may be a gap in expertise: the pelvic surgeon may not be proficient with lumbosacral stabilization and decompression techniques, or the spine surgeon may not be familiar with pelvic ring fixation procedures. In such instances, a close and well-coordinated collaboration between the spine and pelvic surgeon is vital to ensure optimal surgical treatment and to prevent an unnecessary delay of treatment [15–17].

Key clinical factors influencing early clinical decision making and timing of surgical intervention include the patient’s physiological status, the presence of concomitant potentially life-threatening injuries, the presence and severity of neurological deficits, the degree of displacement and instability depending on the sagittal and coronal SPDI pattern [5], and the extent of soft tissue compromise in the lumbosacral and gluteal regions. Similarly, the selection of the most appropriate surgical stabilization method should consider all these factors, rather than focusing solely on the degree of displacement and instability. Each method has its own set of advantages and limitations, including biomechanical stability, feasibility of achieving adequate reduction, and associated risks such as soft tissue trauma [9, 10]. These aspects should be carefully weighed in the context of the individual patient’s clinical situation to determine the most effective treatment while minimizing the risk of complications. Furthermore, the surgical sequence may need to be adjusted based on additional factors, such as the presence of a concomitant displaced anterior pelvic ring injury or acetabular fracture, which can influence the overall treatment strategy.

Current classifications systems for injury patterns associated with TLSI primarily focus on fracture characteristics, degrees of instability, or injury mechanisms; however, they often do not adequately consider these clinical factors. To address this gap, Lehman et al. [18] developed the Lumbosacral Injury Classification System (LSICS), which incorporates clinical factors into the classification of complex lumbosacral injuries. LSICS evaluates three key injury characteristics: injury morphology, posterior ligamentous complex integrity and the patient’s neurological status. In addition, it incorporates three clinical modifiers: systemic injury load and physiological status of the polytraumatized patient, soft-tissue status and expected time to mobility. This comprehensive framework informs clinical decision-making through a proposed treatment algorithm applicable to lumbosacral dislocation injuries, lumbosacral dissociation injuries as well as high-energy vertically unstable sacral fractures, encompassing all injury patterns associated with TLSI. While decision-making remains highly individualized in these complex cases, LSICS may offer a valuable tool to assist clinicians in managing the complexities of TLSI.

## Nonoperative treatment

Despite the lack of robust scientific evidence, nonoperative treatment of TLSI and its underlying conditions is generally regarded as inadequate and typically does not yield satisfactory outcomes.

Nonoperative treatment of lumbosacral dislocation injuries (LSDI) has been reported to result in progressive deformity and low back pain as well as secondary neurological deterioration [2, 11, 19–28]. Reports of acceptable to good outcome following conservative management are exceedingly rare and primarily historical [2, 29–31].

Similarly, nonoperative treatment of high-energy spinopelvic dissociation injuries (SPDI) may lead to progressive deformity, malunion, sagittal imbalance, persistent pain, prolonged immobilization and progressive neurological dysfunction [4, 18, 32–39]. According to Lehman et al. [18], nonoperative treatment may be considered for a small subset of patients, such as those with a kyphotic deformity less than 20° and absence of sacral canal or foraminal compromise and expected immobility of more than three months. However, the 20° threshold was based primarily on the authors’ experience with combat-related SPDI (where 2 of 2 nonoperatively treated patients with initial kyphosis greater than 20° showed progressive kyphosis and worsening symptoms), and this threshold has not been corroborated by other studies.

Unilateral vertical sacral fractures (UVSF) with ipsilateral lumbosacral facet joint involvement, may not only lead to posterior pelvic ring instability (PPRI) but also result in ipsilateral lumbosacral facet joint instability and thus TLSI. Consequently, these injuries are commonly considered indications for surgical fixation [40–45]. However, follow-up data is scarce, and only one study has compared operative versus nonoperative treatment for these injuries [13]. The findings of this recent study challenge the view that all UVSF with lumbosacral facet joint involvement constitute an absolute indication for surgery. It suggests that selected cases, such as non- or minimally displaced injuries with a stable anterior pelvic ring, may be treated nonoperatively. Nonetheless, these findings require validation through larger studies, and the precise criteria for nonoperative treatment have yet to be established (see also “Surgical management of unilateral vertical sacral fractures (UVSF) with ipsilateral L5/S1 facet joint involvement (Type 3 TLSI)” section).

Bilateral complete vertical sacral fractures without a transverse fracture component (BVSF) constitute 61C3.3 pelvic ring injuries according to the 2018 AO/OTA classification. There is widespread consensus that this injury pattern necessitates surgical stabilization [46–48].

## Operative treatment

### Surgical management of lumbosacral dislocation injuries (LSDI; Type 1 TLSI)

Lumbosacral dislocation injuries (LSDI) constitute the first type of TLSI and are characterized by the traumatic disruption of the axial skeleton at the level of the lumbosacral motion segment. These injuries encompass disco-ligamentous and/or bony disruptions of the L5/S1 spinal motion segment, ranging from unilateral facet dislocation to complete dislocation of L5 on S1, the latter commonly referred to as traumatic lumbosacral spondyloptosis. LSDI represent three-column injuries that exhibit acute translational instability.

**Fig. 1:**
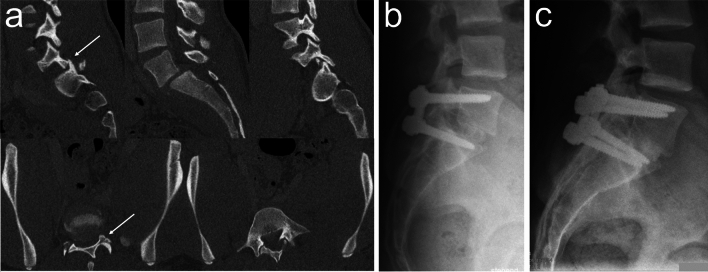
Lumbosacral dislocation injury (LSDI; Type 1 TLSI). A 16-year-old polytrauma patient sustained an LSDI following a skiing collision with a tree and presented with left-sided L5 radicular pain, sensory deficit, and mild L5 motor weakness (Grade 4/5). **a** Preoperative CT images demonstrating a fracture-dislocation of the left lumbosacral facet joint (*white arrows*), anterolisthesis of L5 on S1, and a fracture of the right S1 facet. **b** Postoperative lateral radiograph after L5-S1 instrumentation and circumferential fusion using autologous monocortical bone grafts and cancellous bone harvested from the posterior iliac crest. **c** Lateral radiograph at one-year follow-up. The patient reported no complaints and had already returned to skiing

The authors of larger case series and reviews on LSDI consistently emphasize that surgical stabilization is indicated for all types of LSDI [2, 11, 16, 23–27, 31, 49–51]. They further agree that open reduction, decompression, posterior instrumentation and fusion are the methods of choice. In most cases, posterior instrumentation can be confined to L5 and S1 (Fig. 1). However, in highly unstable LSDI, such as traumatic lumbosacral spondyloptosis, the instrumentation may need to be extended to the Ilium and L4. Other indications for extending instrumentation to L4 include L5 pedicle or vertebral body fractures, as well as concomitant L4/L5 facet joint or disc involvement [24, 27, 51].

The primary *goals* of operative treatment are to restore normal spinal alignment, achieve neurological decompression, and re-establish lumbosacral stability through instrumentation and fusion.

The *timing* of surgical intervention is determined by the extent of neurological injury, associated injuries, and the degree of instability (e.g., unilateral facet dislocation vs. spondyloptosis). Reported rates of neurological deficits in LSDI include 27% (3/11 patients) [26], 50% (63/125 patients) [11], 58% (36/62 patients) [31], and 68% (39/57 patients) [49]. Specific rates for bilateral lumbosacral dislocation without fractures and traumatic lumbosacral spondyloptosis have been reported as 86% (6/7 patients) [50] and 80% (8/10 patients) [51], respectively. If neurological deficits are present, there is broad consensus among authors that operative treatment should be initiated as soon as the general patient’s overall condition permits.

Closed *reduction* maneuvers, particularly those involving excessive hyperkyphosis, are not recommended due to the significant risk of further neurological damage. This can arise from extrusion of disc material into the spinal canal, narrowing of the cross-sectional area of the spinal canal – especially if posttraumatic segmental kyphosis is present prior to reduction – and nerve root stretching [2, 23, 24]. Instead, early open reduction and decompression via a posterior approach, facilitated by transpedicular screws, should be performed. The extent of *decompression* should be tailored to the specific injury pattern. For instance, in cases of unilateral facet dislocation without neurological deficit and no evidence of disc disruption, spinal canal compromise, or neuroforaminal compromise on preoperative MRI, access to the spinal canal achieved by removing the usually disrupted ligamentum flavum may be sufficient, provided that the dislocated facet joint can be reduced without the need for (partial) facetectomy. However, if the preoperative MRI reveals disc disruption or spinal canal or neuroforaminal compromise due to bone fragments, disc material or hematoma – or if MRI is unavailable – it is advisable and safer to decompress the thecal sac and nerve roots prior to reduction. This allows for exploration of the spinal canal, thecal sac, nerve roots, and injured disc both before and after reduction. Moreover, irreducible facet joint dislocation may necessitate at least partial facetectomy. Consequently, decompression may involve partial or complete facetectomy, as well as laminotomy of the inferior and superior aspects of the L5 and S1 laminae, respectively, or even complete laminectomy. Nevertheless, it should be kept in mind that unnecessarily excessive decompression can destabilize the lumbosacral motion segment, whereas at least partially preserving lumbosacral facet joints can help counteract anterior redislocation, decrease the risk of fixation failure, and facilitate posterior fusion [2, 23, 27]. Additionally, dural tears may be encountered, requiring watertight repair, whether by suture or patching techniques. Finally, it should be noted that reduction can become much more difficult if treatment is significantly delayed [2, 23].

*Fusion* in LSDI has been a subject of considerable debate over the past decades, particularly regarding whether posterior/posterolateral fusion (PF) or circumferential fusion, including L5/S1 interbody fusion (IF), should be preferred. Early on, several authors recognized L5/S1 disc injury as a key factor influencing this decision [23, 25, 26, 49]. Aihara et al. [49] and Robertson et al. [25] pointed out that PF alone is insufficient in the presence of disc injury, as it can lead to progressive disc height loss and fixation failure. In contrast, IF can mitigate these complications and save fusion levels. Vialle et al. [26] further emphasized that even a moderate slip of L5 on S1 signifies a significant disc injury and advocated for preoperative MRI evaluation of the disc in all neurologically intact patients. In patients presenting with severe neurological deficits that necessitate emergency decompression, intraoperative exploration of the L5/S1 disc was recommended, and IF was generally advised, as decompression often involves substantial posterior bone resection in these patients. Consequently, only less severe LSDI, such as unilateral facet dislocations, were considered suitable for PF alone, provided that preoperative MRI confirmed disc integrity. In contrast, cases with disc injury should receive IF to ensure optimal outcomes. These recommendations were supported by a recent comprehensive review of 125 cases of traumatic lumbar spondylolisthesis, 74% (93/125) of which involved LSDI [11]. The analysis reported an overall fusion rate of 74%, with IF demonstrating a significantly higher fusion rate compared to PF (88% vs. 69%, respectively, *p* = 0.012). Subgroup analysis revealed that this difference was even more pronounced in patients with disc injuries (87% vs. 46%, respectively, *p* = 0.006), whereas in those without identified disc injuries, the difference was not statistically significant (89% vs. 74%, respectively). Based on these findings, the authors recommended posterior instrumentation with PF alone only for low-grade traumatic spondylolisthesis with an intact disc, while disc injury and high-grade spondylolisthesis necessitate circumferential fusion, including IF. In most cases, performing IF via the same posterior approach used for decompression is the most practical and effective option. However, anterior IF, which allows for a larger and more lordotic interbody device, may also be considered in certain situations, particularly in severely unstable cases such as traumatic lumbosacral spondyloptosis [51, 52]. While anterior IF may increase construct stability and fusion rate, it requires a two-step procedure or at least patient repositioning from prone to supine.

#### Expected outcomes and complications

The most recent and comprehensive review analyzed 125 cases of traumatic lumbar spondylolisthesis, of which 74% (93/125) were LSDI [11]. The mean age at the time of injury was 30.5 years, with the majority of patients being male, and two-thirds presenting with concomitant extraspinal injuries. Neurological deficits were observed in 50% of patients, while one-quarter had high-grade spondylolisthesis (Grade 3 and 4). All but 8 patients were treated operatively. Regarding outcomes, both pain and neurological deficits significantly improved at the final follow-up, which occurred at an average of 27.2 ± 26.4 months (range 1–120 months). At follow-up, 94% of patients were ambulatory, 20% experienced residual pain, and 25% had persistent neurologic deficits, including weakness, numbness, or disturbances in bladder and bowel function. In patients with disc injuries, but not in those without, the fusion rate (87% vs. 46%) and proportion of ambulatory patients (100% vs. 83%) was significantly higher following IF compared to PF alone. The overall complication rate was 22% (28/125 patients), with complications occurring after an average of 13.3 ± 23.6 months (range 0–96 months). The most common complications included implant failure (39%), progression of spondylolisthesis following nonoperative treatment or posterior decompression alone (18%), and infection (14%).

### Surgical management of spinopelvic dissociation injuries (SPDI; Type 2 TLSI)

Spinopelvic dissociation injuries (SPDI) constitute the second type of TLSI. This injury pattern is characterized by the unique combination of a high transverse sacral fracture (occurring at or above the S2/S3 junction) and bilateral vertical sacral fractures. The transverse sacral fracture component is most commonly located at the S1/S2 junction or within the S2 vertebral body [3–5], while the bilateral vertical sacral fractures are typically transforaminal and may also extend medially to the foramina [53]. This multiplanar sacral fracture pattern mechanically separates the spine from the pelvis at the level of the sacral bone, leading to TLSI. The U-shaped pattern is the most frequent coronal fracture variant (46% according to [12]) and results solely in TLSI, whereas the H-, Y- and lambda-shaped coronal fracture patterns lead to both TLSI and posterior pelvic ring instability (PPRI).

The *goals* of operative treatment for SPDI are to decompress neural structures in cases of encroachment, achieve reduction and restore physiological alignment—particularly of the central upper sacrum fragment—and re-establish lumbosacral and posterior pelvic ring stability through reliable fixation, facilitating early full weight-bearing [43, 54].

*Timing* of operative intervention in SPDI depends on the extent of neurological injury and the degree of fracture displacement and instability. In many cases, however, the patient’s physiologic status dictates the timing of surgery, as SPDI patients are often multiply injured [6, 55]. This likely contributes to the considerable average time to surgery observed in two reviews on SPDI, despite the high incidence of neurological deficits (80% and 68%, respectively): 5 days (range 0–45 days) and 8.6 days (range 0–43.5 days) [6, 12]. With increasing delay, however, achieving reduction becomes progressively more challenging, if not impossible, and neurological recovery may be negatively affected, although the latter is still a topic of debate, and its impact remains largely unclear. Therefore, if the patient cannot tolerate early, extensive surgery – such as open decompression and reduction – and the injury pattern is amenable to closed reduction, closed reduction should be considered.

*Decompression* of encroached neural structures can be categorized as either direct or indirect. Direct decompression of sacral nerve roots is achieved via sacral laminectomy, with or without foraminotomy. This procedure alleviates sacral canal narrowing, enables the removal of free bone fragments within the sacral canal or foramina impinging on nerve roots, and permits the anterior repositioning of retropulsed bone fragments using angled impactors. In contrast, indirect decompression is achieved through fracture reduction, particularly of angular and translational displacement of the upper central sacrum, and improves or restores sacral canal patency without laminectomy. Indirect decompression is particularly important in SPDI, where fracture displacement usually represents the main contributor to nerve root compression or traction. Consequently, sacral nerve root decompression is typically not successfully achieved when there is significant residual fracture malreduction. The optimal timing for decompression and the role of sacral laminectomy remain controversial and not well understood [38, 56–62]. A review by Kepler et al. [56] found no clear benefit of early decompression within 72 h in sacral fractures and reported no difference in neurologic recovery between formal laminectomy and indirect decompression. Another review observed no difference in neurological recovery between operative and nonoperative treatment of low and high transverse sacral fractures [57]. However, these findings should be interpreted with caution and are insufficient to inform clinical decision making, as the analyzed studies were of low methodological quality and likely subject to treatment selection biases. We agree with several authors of recent articles [15, 16, 33, 37, 38], that neurological decompression should be performed as soon as the patient’s physiological status allows. For the majority of displaced SPDI cases, we also prefer a combination of direct and indirect decompression, although sacral laminectomy may be omitted in cases where indirect reduction sufficiently restores sacral canal patency and access to the upper central sacrum is not needed for direct reduction maneuvers.

*Reduction* of the central upper sacrum fragment is a critical and usually the most challenging step in the operative treatment of displaced SPDI. The primary goals of reduction are twofold: restoring physiological spinal alignment and achieving indirect decompression of the sacral canal. It can be categorized into closed vs. open reduction, as well as direct vs. indirect reduction. Direct reduction implies direct manipulation of the upper central sacrum, while indirect reduction employs techniques that facilitate re-alignment without direct access to the fracture site. A comprehensive discussion of strategies for achieving adequate reduction is beyond the scope of this article and has been addressed elsewhere [4, 5, 37, 43, 54, 62–69]. Briefly, techniques for direct reduction may include a threaded Schanz pins placed into the upper sacral body, an elevator or lamina spreader to disimpact the transverse fracture component, or a bone impactor to directly reduce the apex of angulation, while indirect reduction techniques may involve closed reduction maneuvers, patient positioning, bilateral supracondylar traction, femoral distractors, and manipulation via the screws and rods of the lumbopelvic instrumentation, such as distraction along the rods.

It is crucial to emphasize that angular and translational displacement of the upper central sacrum significantly impacts *sagittal alignment and balance* of the entire spine. In particular, the kyphotic angulation of the upper central sacrum in Type 1 and Type 2 SPDI results in an increase of sacral slope and pelvic incidence (PI), with the latter being a fixed anatomical parameter with only minor positionally-dependent changes in the absence of fracture [70, 71]. As native PI and lumbar lordosis (LL) are closely related, the patient’s native LL will be too low relative to the traumatically increased PI, resulting in a PI-LL mismatch and anterior translation of the axis of gravity [34, 72]. Segmental, regional and global compensatory mechanisms, such as lumbar hyperextension, pelvic retroversion and thoracic hyperextension, can contribute to limit the consequences of a traumatically increased PI and to maintain sagittal balance of the spine above the pelvis (compensated balance), but may lead to adverse effects, such as increased stresses on posterior lumbar structures, increasing the risk for accelerated facet joint osteoarthritis, Baastrup disease and compensatory discopathy [73, 74]. With increasing severity of sacral kyphosis, the increased PI can no longer be compensated for, resulting in sagittal imbalance and difficulty with maintaining an upright stance. Insufficient surgical restoration of spinopelvic alignment has been shown to negatively affect outcomes in degenerative spinal disorders [75, 76]. In accordance with these findings, Lee et al. [34] demonstrated that SPDI patients with poorer global sagittal alignment (sagittal vertical axis (SVA) ≥ 4 cm vs. SVA < 4 cm) experience significantly worse clinical outcomes, including increased bodily pain and higher Oswestry Disability Index scores. Similarly, Ruatti et al. [61] found that lumbo-pelvic mismatch was associated with poorer functional outcomes, defined by a Majeed score below 75. Additionally, Lindahl et al. [5] reported that patients with smaller residual postoperative translational and angular displacement of the upper central sacrum had better clinical outcomes. Establishing a specific threshold value for acceptable kyphotic angulation that does not adversely impact clinical outcomes would be desirable when balancing the risks and benefits of different treatment strategies with varying potentials for reduction. However, such a threshold remains undefined due to the lack of long-term outcome studies, and defining absolute values is challenging as these also depend on each patient's specific spinopelvic parameters and compensatory capacities. It is also important to note that corrective surgery of SPDI malunions is very complex and generally associated with worse outcomes [4, 43]. Overall, adequate reduction, although often difficult to achieve, is critical to prevent sagittal imbalance after SPDI. In U-, Y-, and lambda-shaped SPDI, adequacy of sagittal plane reduction of the central upper sacrum can be intraoperatively checked using the posterior and anterior cortex of the caudal central sacrum fragment as a reference [77]. However, in H-shaped SPDI the caudal central fragment is loose and no longer a reliable reference. In this scenario, PI can be determined intraoperatively on lateral fluoroscopic images and used to assess adequacy of sagittal plane reduction. Since the preinjury PI of a specific patient is usually unknown, normal values for PI can be used as a target for reduction [72]. However, these reported norms vary across populations, with substantial ranges within specific groups (pooled mean PI: 50.6°, optimal range: 39°–62°) [78]. Therefore, in our practice, we prefer to approximate an individual patient’s PI using predictive formulas for LL as a function of PI, with LL measured on preoperative computed tomography in supine position [73, 79, 80].

**Fig. 2:**
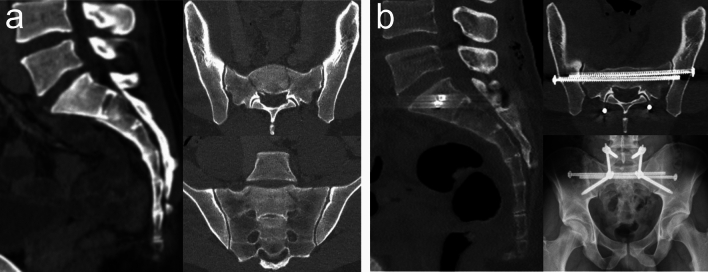
Spinopelvic dissociation injury (SPDI; Type 2 TLSI). A 29-year-old male paraglider sustained a SPDI with sacral nerve root dysfunction (hypoesthesia and impaired sharp-dull discrimination in the S2-S5 dermatomes but preserved voluntary anal contraction) and a right-sided open lower leg shaft fracture following a fall from height. **a** Preoperative CT images showing a U-shaped Type 2a SPDI with the transverse fracture component at S2. **b** Postoperative CT images and an AP radiograph after closed reduction in the prone position and percutaneous TLPF demonstrate anatomic reduction

**Fig. 3:**
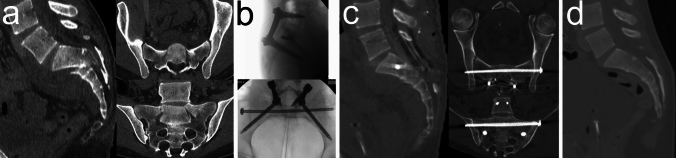
Spinopelvic dissociation injury (SPDI; Type 2 TLSI). A 19-year-old patient sustained an SPDI with cauda equina dysfunction (Gibbons Grade 4) following a suicidal jump. **a** Preoperative CT images showing a U-shaped Type 2b SPDI with the transverse fracture component at S2. **b** Intraoperative fluoroscopic images after open decompression (including sacral laminectomy), reduction, and TLPF (with S2 alar-iliac screws). **c** Postoperative multiplanar CT reconstructions demonstrating realignment of the central upper sacrum compared with **d** a preinjury CT scan available for this patient

The *three primary methods for stabilizing SPDI* are triangular lumbopelvic fixation (TLPF), lumbopelvic fixation (LPF), and sacral screw fixation [6, 12, 43, 54]. The *three main treatment strategies* are as follows:

- Percutaneous in situ fixation using either percutaneous sacral screw fixation or percutaneous TLPF, which does not involve decompression [3, 81]
- Closed reduction followed by percutaneous fixation, which includes indirect decompression provided that closed reduction is successful at least to some extent [61, 63, 82] (Fig. 2)
- Open reduction, decompression, and TLPF or LPF, which often entails both direct and indirect decompression [4, 5, 60, 83] (Fig. 3)

Preoperative decision-making involves selecting the most appropriate treatment strategy based on the outlined treatment goals while considering factors such as the patient’s physiological and neurological status, the sagittal and coronal fracture patterns, and the condition of lumbosacral and gluteal soft tissues. Each stabilization method and treatment strategy presents its own set of advantages and drawbacks, which must be carefully evaluated and tailored to the individual patient.

*Percutaneous screw fixation* using iliosacral (IS) or transsacral (TS) screws offers advantages such as minimal soft tissue trauma, relatively short operative time, and negligible blood loss. However, this minimally invasive technique provides fixation only perpendicular to the weight-bearing axis, and the stability of iliosacral screws may be substantially compromised in cases of sacral comminution (see also “Surgical management of unilateral vertical sacral fractures (UVSF) with ipsilateral L5/S1 facet joint involvement (Type 3 TLSI)” and “Surgical management of bilateral vertical sacral fractures without a transverse fracture component (BVSF; Type 4 TLSI)” sections). Transsacral screws, also referred to as transiliac-transsacral screws, are generally considered to offer enhanced fixation but critically rely on the availability of a safe bone corridor to accommodate at least two screws cranially to the transverse fracture component [84]. Moreover, weight-bearing has been typically restricted for 6 to 12 weeks following isolated sacral screw fixation [3, 81].

*Bilateral triangular lumbopelvic fixation (TLPF) and lumbopelvic fixation (LPF)* are the most common techniques for stabilizing SPDI, used in 69% of cases reviewed by Bäcker et al. [6]. These stabilization techniques provide superior biomechanical stability to sacral screw fixation alone and enable early full weight-bearing [85–88]. However, compared to percutaneous sacral screw fixation, open TLPF or LPF are associated with longer operative times, greater blood loss, and higher rates of soft tissue complications and infections [89, 90]. In cases where open reduction or decompression is not required, percutaneous TLPF may be employed to minimize soft tissue complications commonly associated with open techniques [65, 91], whereas LPF via minimal skin incisions may be less feasible due to the need for transverse connector insertion. Alternatively, percutaneous TLPF or LPF can be used along with open reduction to minimize the extent of soft tissue dissection and exposure required specifically for open screw insertion, using only the amount of dissection necessary for the decompression and fracture reduction.

The *TLPF* construct typically consists of vertically oriented bilateral L5 to ilium instrumentation, complemented by horizontally oriented transsacral (or iliosacral) screw fixation to address the multidirectional instability characteristics in SPDI (see also “Surgical management of unilateral vertical sacral fractures (UVSF) with ipsilateral L5/S1 facet joint involvement (Type 3 TLSI)” and “Surgical management of bilateral vertical sacral fractures without a transverse fracture component (BVSF; Type 4 TLSI)” sections). The vertically orientated components primarily prevent caudal migration of the central cranial sacrum—particularly important in H-shaped SPDI—while also counteracting the flexion moment. The horizontally oriented component prevents splaying of the vertical sacral fractures and enhances rotational stability across the unstable spinopelvic junction. This configuration effectively offloads the multiplanar sacral fracture, transferring load from the lumbar spine through the iliac wings to the hip joints. Biomechanical data from synthetic bone models suggest that extending instrumentation to L4 is not necessarily advantageous, whereas a horizontally oriented transsacral screw fixation is critical to provide sufficient fixation [92]. However, extension to L4 is typically required in Type 3b SPDI (see below) and may also be necessary in cases with a high native sacral slope and pronounced lordosis of the lower lumbar spine [93]. Additionally, S1 screws may be inserted to facilitate reduction—provided they do not cross a vertical fracture, which could impede reduction—and to enhance construct stability [94].

Lumbopelvic fixation (*LPF*) is similar to TLPF but does not involve the use of sacral screws. This fixation technique has been also referred to as “modified” TLPF, as it employs a transverse connector (also known as a cross-link) between the rods of the iliolumbar instrumentation instead of a sacral screw [95]. Several authors have successfully applied LPF to stabilize SPDI [5, 6, 93, 95–99]. Lindahl et al. [5, 54], for example, demonstrated its efficacy in H-shaped SPDI. Their preferred lumbopelvic fixation construct consists of two pairs of lumbar pedicle screws in L4 and L5, bilateral longitudinal rods, at least one transverse connector, and two pairs of iliac screws. Their procedure typically begins by attaching the rods to the lumbar pedicle screws and securing a transverse connector to the rods before performing SPDI reduction. In a next step, this lumbar construct together with two pairs of pointed reduction clamps facilitate open reduction of this most complex and unstable SPDI pattern. This approach allows for stepwise or simultaneous correction of cranial and lateral displacement of the hemipelves at the vertical fracture components, sagittal translational and angular displacement of the upper central sacrum at the transverse fracture component, and displacement of the caudal central sacrum fragment. Once reduction is achieved, iliac screws are placed and connected to the distal ends of the rods via offset connectors. Few authors have utilized bilateral iliolumbar instrumentation without any horizontally oriented fixation, such as a sacral screw or a transverse connector, to stabilize high-energy SPDI [45, 100, 101]. From a biomechanical perspective, this construct provides reduced stability in lumbopelvic fixation and may not sufficiently counteract the splaying of vertical fracture components in coronal SPDI patterns other than U-shaped ones under axial loading [87, 92].

*Concomitant anterior pelvic ring injuries* are often encountered in SPDI [4, 5, 55]. If an associated displaced anterior pelvic ring or acetabular fracture requires reduction and fixation, starting with an anterior approach is strongly recommended. This facilitates the reduction of vertical sacral fractures in H-, Y- and lambda patterns and prevents irreducible anterior pelvic ring malreduction, which can be especially problematic after rigid TLPF or LPF.

Depending on the type of sagittal and coronal fracture patterns, different challenges and priority issues arise in the management of SPDI.

#### Type 1 and Type 2 SPDI

In Type 1 SPDI, the central upper sacrum exhibits kyphotic angulation without translational displacement. This sagittal pattern is less frequently observed after high-energy trauma compared to Type 2 SPDI [3, 96, 102]. Since there is no posterior translation of the central upper sacrum, the sacral canal remains uncompromised. However, neurological deficits may still occur due to neuroforaminal compromise or stretching of the nerve roots, particularly L5 and S1. In Type 2 SPDI, the central upper sacrum demonstrates both kyphotic angulation and posterior translation. Similar to Type 1, there is a need for reduction of significant kyphotic angulation; however, Type 2 also necessitates sacral canal decompression if neurological deficits are present due to neural encroachment. This decompression can be achieved through indirect or direct methods. Type 1 and Type 2 SPDI encompass a broad spectrum of injuries characterized by varying degrees of displacement and different coronal fracture patterns.

In non- or minimally displaced SPDI without neural encroachment, *percutaneous in situ screw fixation* is a viable option, particularly for U-shaped coronal fracture patterns. U-shaped SPDI lead to TLSI without PPRI, and the intact lower sacrum resists caudal migration of the central upper sacrum. In this less complex instability pattern, the primary goal of sacral screw fixation is to prevent progressive kyphotic angulation, while the limited ability of sacral screws to resist shear forces is less significant in these cases. To ensure adequate stability, a minimum of two transsacral screws positioned cranial to the transverse fracture component (typically in S1) is generally considered necessary [82, 84, 103, 104]. Postoperative protocols often recommend weight-bearing restrictions for 6 to 12 weeks when relying solely on sacral screw fixation, as reported in the literature [3, 63, 67, 81]. The addition of percutaneous L5 to ilium instrumentation can avoid prolonged immobilization and may be particularly indicated in cases where sacral dysmorphism precludes secure placement of transsacral screws above the transverse fracture component. Advantages of percutaneous in situ sacral screw fixation include minimal soft tissue trauma, negligible blood loss, short operative time, no need for prone positioning of the multiple injured patient, and low complication rate. As reduction and decompression is not involved in this procedure, this technique is only suitable for a small subset of SPDI without significant displacement and not requiring decompression. The main challenge in determining the indication for in situ fixation is the lack of knowledge about what constitutes an acceptable angular displacement of the upper central sacrum and more specifically, what degree of residual kyphotic deformity does not adversely affect clinical outcomes. A few retrospective studies have reported outcomes following in situ sacral screw fixation for high-energy SPDI: Nork et al. [3] utilized long, fully threaded 7.0-mm iliosacral screws to traverse both vertical fractures with each screw in 13 patients with U-shaped SPDI (one Type 1, eight Type 2 and four Type 3 fractures according to Roy-Camille). Among these patients, nine presented with preoperative neurological deficits without neural encroachment. The mean duration of surgery was 48 min, with negligible blood loss, and no complication was observed, except for a single case of incomplete screw disengagement causing buttock pain. All fractures healed without changes in alignment. Postoperatively, all patients were immobilized for 2 to 3 months using a brace and wheelchair. Moo Young et al. [81] employed iliosacral screws or transsacral screws in a multicenter series of 53 displaced SPDI (94% Type 2 and 6% Type 3), including 7 cases with Gibbons Grade 2 or Grade 3 neurological deficits. Complications were rare, with one case of nonunion and two cases of iliosacral screw loosening that went on to uneventful union. Postoperative weight-bearing protocols varied, with periods of non-weight-bearing ranging from 6 to 12 weeks. Helgeson et al. [32] described 15 combat-related SPDI cases, 7 of which were treated with iliosacral screw fixation, though specific outcomes for this subgroup were not reported. Saiz et al. [104] observed no complication following percutaneous screw fixation of 76 cases of mildly displaced (mean kyphotic angulation: 14.2° ± 8.6°) U-shaped SPDI, but this series included only 2 high-energy injuries. While these studies reported complications and short-term neurological recovery, none included clinical outcome data or postoperative assessments of sagittal balance. In our view, a significant portion of SPDI cases in the series of Nork et al. and Moo Young et al. cannot be considered minimally displaced and may therefore be unsuitable for in situ screw fixation, given their mean preoperative kyphosis values of 29° (range 6° to 75°) and 30° (range 0° to 70°), respectively. These values fall within the range of those associated with poor clinical outcomes in the study by Lindahl et al. [5], where patients with poor clinical outcomes had significantly greater residual postoperative kyphosis than those with good outcomes (29° ± 15° vs. 17° ± 10°, respectively).

Significantly displaced Type 1 and Type 2 SPDI, with or without neurological involvement, require reduction and decompression. Reduction and indirect decompression may be achieved through *closed reduction* maneuvers in either the prone or supine position, particularly in patients with a U-shaped coronal fracture pattern. Ruatti et al. [63] described a closed reduction technique for Type 2 SPDI, followed by percutaneous iliosacral screw fixation. In their technique, the intubated patient is positioned supine, with multiple rolled sheets placed under the lumbosacral region to induce hyperlordosis. Following neuromuscular blockade, a strong manual traction force is applied through bilateral distal femoral traction pins under lateral fluoroscopic control, while two assistants provide manual countertraction at the armpits. This method achieved remarkable reductions in three patients documented by the authors, highlighting its potential effectiveness for Type 2 SPDI. In a subsequent study by the same group [61], the outcomes of this closed reduction technique were evaluated in 20 cases of Type 2 SPDI, including 10 U-variants, 9 H-variants, and 1 Y-variant. The mean preoperative PI and posterior translational displacement improved from 61° to 49° and from 64 to 6%, respectively. Complications were limited to two malpositioned iliosacral screws. After a mean follow-up period of 42.4 months (range: 12–121 months), the mean VAS pain score was 0.87/10 and the mean Majeed score was 86.6. A lumbopelvic mismatch was observed in three patients (15%), and it was associated with poorer outcomes, defined as Majeed scores below 75. Among the 10 patients with neurological deficits (5 Grade 2, 1 Grade 3, and 4 Grade 4 according to Gibbons et al. [105]), 7 achieved complete neurologic recovery, while 2 demonstrated partial recovery. Irifune et al. [82] also employed a reduction technique performed in hyperextended supine position in 16 consecutive patients (12 Type 2 and 4 Type 3 SPDI), followed by percutaneous screw fixation using transsacral screws (n = 13) or iliosacral screws alone (n = 3). The kyphosis of the upper central sacrum was improved from 39° ± 21° preoperatively to 21° ± 7° postoperatively, while translational displacement was reduced from 8.7 ± 8.0 mm to 2.8 ± 4.0 mm. Five patients required a second-stage sacral laminectomy due to remnant bone fragments in the sacral canal, identified on postoperative CT imaging. Complications included screw loosening (n = 3) and screw malposition (n = 4). After a mean follow-up of 28 months (range: 12–71 months), a significant loss of reduction was observed, with a final mean kyphosis of 27.9° ± 13.0° (range: 13°–60°). Notably, one patient with bilateral partially threaded iliosacral screw fixation in S1 and S2 experienced more than 30° of reduction loss. Clinical outcomes were classified as good in 73% of patients and poor in 27%, according to the German Multicenter Study Group Pelvis Outcome Scale (mean score: 2.93 ± 1.28). Neurological improvement of at least one Gibbons grade was observed in 8 of 12 patients (67%) with initial neurological deficits, with partial recovery in 3 patients and complete recovery in 5. Furthermore, two other authors have described percutaneous reduction maneuvers using Schanz pins [67, 106]. In our practice, we prefer closed reduction in the prone position, with the traction force directed along the long axis of the central upper sacrum. Based on our experience, which aligns with the findings of Ruatti et al. [61, 63], successful closed reduction critically depends on two key factors: a short interval between injury and reduction, and the use of distal femoral traction pins to generate a sufficiently intense traction force to enable disimpaction of the transverse fracture component and reduction of the upper central sacrum. The advantages of closed reduction and percutaneous fixation are similar to those of percutaneous in situ screw fixation, with the added benefit of enabling indirect reduction and decompression. However, this approach has its limitations. Only a subset of Type 1 and Type 2 SPDI cases are suitable for closed reduction, and closed reduction does not always achieve satisfactory reduction and decompression. In our view, U-shaped Type 1 and Type 2 SPDI without significant comminution and neural encroachment caused by free bone fragments in the sacral canal or neuroforamina are particularly well-suited for this technique. In contrast, H-, Y-, and lambda-shaped variants should only be considered for closed reduction if their vertical fracture components are nondisplaced. When vertical fracture components are significantly displaced or unstable, closed reduction maneuvers may exacerbate displacement, stretch nerve root, and potentially result in iatrogenic neurological injury. Furthermore, associated injuries such as femoral fractures or unstable spinal fractures, as well as other factors like severe obesity, can impede closed reduction attempts. Despite these limitations, closed reduction remains an appealing option for a subset of Type 1 and Type 2 SPDI patients, particularly those unable to tolerate early extensive surgery or prolonged prone positioning.

*Open reduction, decompression, and bilateral TLPF or LPF* are the most commonly applied treatments for SPDI [6] and remain the preferred treatment strategy for many authors [15, 16, 33]. High-energy Type 2 SPDI are often characterized by severe displacement and comminution. This approach allows for both indirect and direct reduction and decompression and is considered the method of choice particularly in the following scenarios: (1) displaced vertical fracture components; (2) comminuted transverse or vertical fracture components with neural encroachment caused by loose bone fragments within the spinal canal or neuroforamina; (3) significant residual displacement or persistent neural impingement after closed or percutaneous indirect reduction attempts; and (4) inadequate kyphosis correction through indirect methods, requiring direct manipulation of the upper central sacrum, such as with Schanz pins. In addition, fusion of disrupted lumbosacral facet joints requires an open approach. L5/S1 facet joint involvement has been reported in up to 46% of SPDI [14, 60], and nonfusion of facet joint injuries has been associated with poorer outcomes, including persistent pain [60]. The two main disadvantages of open reduction, decompression, and TLPF or LPF are prolonged operative time and an increased risk of wound complications (see below).

#### Type 3 SPDI

Type 3 SPDI are characterized by anterior translational displacement of the upper central sacrum relative to the lower sacrum in the sagittal plane. Unlike Type 2 SPDI, where nerve roots are typically compressed, nerve roots in Type 3 SPDI are generally stretched rather than compressed. In cases of complete displacement (classified as Type 3b according to Lindahl et al. [5]), the upper central sacrum typically slips down in front of the caudal sacrum, rendering the L5 pedicle screw entry points inaccessible. The primary objectives in managing these fractures are to restore sacral length and to reduce anterior translational displacement. Type 3 SPDI, especially those with complete translational displacement and high instability (Type 3b), are generally unsuitable for closed reduction. Closed reduction techniques fail to achieve the necessary combination of distraction and posterior translation and carry a significant risk of iatrogenic nerve root injury due to excessive nerve root traction. Consequently, open reduction combined with TLPF or LPF is considered the preferred treatment approach for Type 3 SPDI. Reduction is typically achieved in a stepwise manner as described by Starantzis et al. [66], Linhart et al. [68], and Lindahl et al. [5]: In a first step, sacral length is restored via distraction along the rod of a L4 to ilium instrumentation, which also provides access to the L5 vertebra for pedicle screw insertion. In a second step, anterior translational displacement is gradually reduced by tightening persuaders attached to the pedicle screws. Sacral laminectomy is often performed during the procedure to allow direct visualization and control of the sacral canal and nerve roots. Studies on percutaneous stabilization of SPDI, including sacral screw fixation as well as percutaneous TLPF or LPF, have included only a limited number of Type 3 SPDI cases. Furthermore, these studies often lack detailed descriptions of fracture characteristics and specific outcome data for this subgroup [3, 65, 81, 107].

#### Expected outcomes and complications

Reported outcomes after SPDI vary depending on the specific populations and injury patterns studied. At presentation, 68% to 80% of patients with SPDI have neurological deficits [6, 12]. A review of 50 studies, covering 379 SPDI cases between 1969 and 2018, found an overall neurological improvement rate of 65%, though 53% of patients with neurological deficits retained some degree of residual neurologic impairment [6]. After closed reduction and percutaneous fixation, Ruatti et al. [61] reported partial and complete neurologic recovery in 90% and 70% of 10 Type 2 SPDI cases with neurological deficits (5 Grade 2, 1 Grade 3, and 4 Grade 4 according to Gibbons et al. [105]). Neurological recovery following open reduction, decompression, and TLPF or LPF has been documented by multiple authors. In a series of 18 patients with Type 2 to Type 4 SPDI and cauda equina dysfunction (i.e. Gibbons Grade 4), Schildhauer et al. observed complete recovery of bowel and bladder function in 56% (10/18) and complete neurological recovery in 33% (6/18). They found that complete neurological recovery was significantly associated with both the absence of sacral nerve root disruption and incomplete cauda equina syndrome. Among 11 patients with at least one disrupted sacral nerve root, 36% (4/11) still regained full bowel and bladder function, likely due to the redundancy and bilaterality of bowel and bladder innervation. Similarly, Ayoub [60] observed significantly better neurological recovery in patients with incomplete versus complete cauda equina dysfunction. In his series, only one patient in the complete group had persistent bowel and bladder impairment, while full neurological recovery was achieved in 77% (13/17) of patients in the incomplete group and 55% (6/11) in the complete group. Lindahl et al. [5] reported complete neurological recovery in 19% (7/36) of patients with H-shaped SPDI, including 7 patients with concomitant spinal injuries and associated neurological deficits. Among those with preoperative Gibbons Grade 4 neurological injuries, 59% (17 of 29) achieved complete recovery of bowel and bladder function. Importantly, the extent of initial translational displacement at the transverse sacral fracture was significantly associated with neurological recovery, with improvement observed in only 31% of patients with complete displacement compared to 75% of those with incomplete displacement.

Regarding functional outcomes, Lindahl et al. [5] found that patients with complete initial translational displacement had worse clinical outcomes than those with partial displacement, while factors such as age, sex, ISS, and Roy-Camille type (2 vs. 3) had no significant impact on outcomes, as measured by the German Multicenter Study Group Pelvis Outcome Scale. More importantly, the quality of reduction, specifically in terms of postoperative residual translational displacement and kyphotic angulation of the central upper sacrum, was associated with clinical outcomes. Consistent with these findings, other studies have also demonstrated that quality of reduction and restoration of spinopelvic alignment are associated with better outcomes, as mentioned above [34, 61]. Mid- and long-term functional outcomes have been evaluated in only a few studies [83, 102, 108–111], with data showing considerable heterogeneity due to variations in injury morphology, neurological impairment, associated injuries, treatment strategies, and often unspecified quality of reduction. Together, these factors make it difficult to fully understand the overall impact on physical and psychological function. In general, however, most patients continue to experience some degree of impairment, including lumbosacral pain, mobility limitations, and sexual dysfunction.

Bäcker et al. [6] reviewed complications after surgical treatment of SPDI, reporting an overall complication rate of 29% without detailing specific types. Complications related to open TLPF or LPF primarily include iliac screw prominence, wound healing disturbances (with or without infection), and asymptomatic rod breakage after fracture healing [4, 35, 55, 60, 90]. Bellabarba et al. [89] observed wound healing disturbances in 26% of 19 consecutive patients, of whom 2 had open fractures and 10 had Morel-Lavallée lesions. More recent studies reported wound healing complications in 5% to 17% [5, 94, 98, 112] after open and 0% to 6% after percutaneous TLPF or LPF [65, 91, 101]. Additionally, screw loosening, screw breakage, loss of reduction and nonunion have been observed after isolated sacral screw fixation [67, 81, 82].

### Surgical management of unilateral vertical sacral fractures (UVSF) with ipsilateral L5/S1 facet joint involvement (Type 3 TLSI)

Unilateral vertical sacral fractures (UVSF) with the proximal fracture line (PFL) running through or medial or both medial and lateral to the ipsilateral S1 facet may not only result in posterior pelvic ring instability (PPRI) but also in ipsilateral lumbosacral joint instability and thus in TLSI. Isler was the first to point out that UVSF with the PFL passing through or medial to the ipsilateral S1 facet have to be clearly distinguished from those with the PFL passing lateral to the ipsilateral S1 facet [113]. In the majority of UVSF, the PFL passes lateral to the ipsilateral S1 facet. Consequently, the ipsilateral S1 facet remains continuous with the medial portion of the sacrum and the integrity of the ipsilateral lumbosacral facet joint remains intact. In such cases, the UVSF may lead to PPRI, but it cannot result in TLSI. However, if the PFL passes through or medial to the ipsilateral S1 facet, the structural continuity of the ipsilateral S1 facet with the medial portion of the sacrum is disrupted. This disruption can compromise the integrity and stability of the ipsilateral lumbosacral facet joint. In this situation, any displacement of the injured hemipelvis (i.e., the hemipelvis lateral to the UVSF) results in subluxation or dislocation of the S1 facet (or a portion of it if the PFL passes through the facet) relative to the L5 facet, leading to facet joint instability and incongruence. In addition, if the PFL is Y-shaped and passes medial as well as lateral to the S1 facet, this leads to a loose S1 facet without continuity to the sacrum. As a consequence, UVSF with the PFL running through or medial or both medial and lateral to the ipsilateral S1 facet may not only result in PPRI but also in TLSI.

The overall *rate* of facet joint involvement in UVSF has been reported in 4 studies and ranges from 9 to 33% [13, 113–115]. Two of these studies also distinguished between vertically unstable and rotationally unstable UVSF and found substantially higher rates in type C (38% and 38%) than in type B injuries (3.5% and 11%) [113, 114]. In a more recent study, Danford et al. [14] identified facet joint involvement in 53 of 476 (11%) patients with pelvic ring injuries, with only 16% documented in radiology reports. Patient-related factors associated with facet joint injury included younger age, high-energy mechanism, and higher ISS. Injury-related factors, amongst others, included Denis zone 2 fractures, Denis zone 3 fractures, bilateral displaced sacral fractures, and L5 transverse process fractures.

It is important to emphasize that ipsilateral L5/S1 facet joint involvement encompasses a *spectrum of patterns* with varying degrees of severity and corresponding effects on lumbosacral facet joint stability. For instance, a noncomminuted, undisplaced UVSF with a simple PFL running medial to the S1 facet, without L5/S1 facet joint subluxation and with an intact facet joint capsule, may have little to no impact on ipsilateral lumbosacral facet joint stability. In contrast, a highly comminuted, significantly displaced UVSF with L5/S1 facet joint dislocation clearly compromises lumbosacral stability. Thus, a detailed analysis of the specific facet joint injury pattern and its impact on lumbosacral stability is crucial for determining the most appropriate treatment strategy.

UVSF with ipsilateral L5/S1 facet joint involvement are generally regarded as indications for surgical fixation, as they are typically considered more severe injuries than UVSF with the PFL passing lateral to the S1 facet, given their potential to compromise lumbosacral stability [40–45, 116, 117]. However, clinical and radiological follow-up data remain scarce, with only two recent studies specifically reporting outcomes for this subgroup of UVSF [13, 118].

Regarding surgical treatment considerations, lumbosacral facet joint involvement in UVSF has a threefold *impact*: First, surgical fixation must address not only PPRI, but also TLSI. Second, a locked L5/S1 facet joint dislocation (Isler Type 2c) significantly alters the reduction strategy and surgical sequence. This pattern typically prevents reduction of the displaced hemipelvis by traction or anterior pelvic ring reduction and fixation. In such cases, an initial open posterior reduction is required. Third, the surgeon has to bear in mind that posttraumatic ipsilateral lumbosacral facet joint degeneration resulting from residual lumbosacral joint instability or incongruence, may contribute to persistent lumbosacral pain and negatively influence outcomes [97, 113–115, 119].

The *aims* of operative treatment for UVSF with ipsilateral facet joint involvement include fracture reduction, restoration of both posterior pelvic ring and lumbosacral stability, facilitation of early weight-bearing, and decompression in cases of neurologic deficits caused by sacral canal or neuroforaminal compromise.

**Fig. 4:**
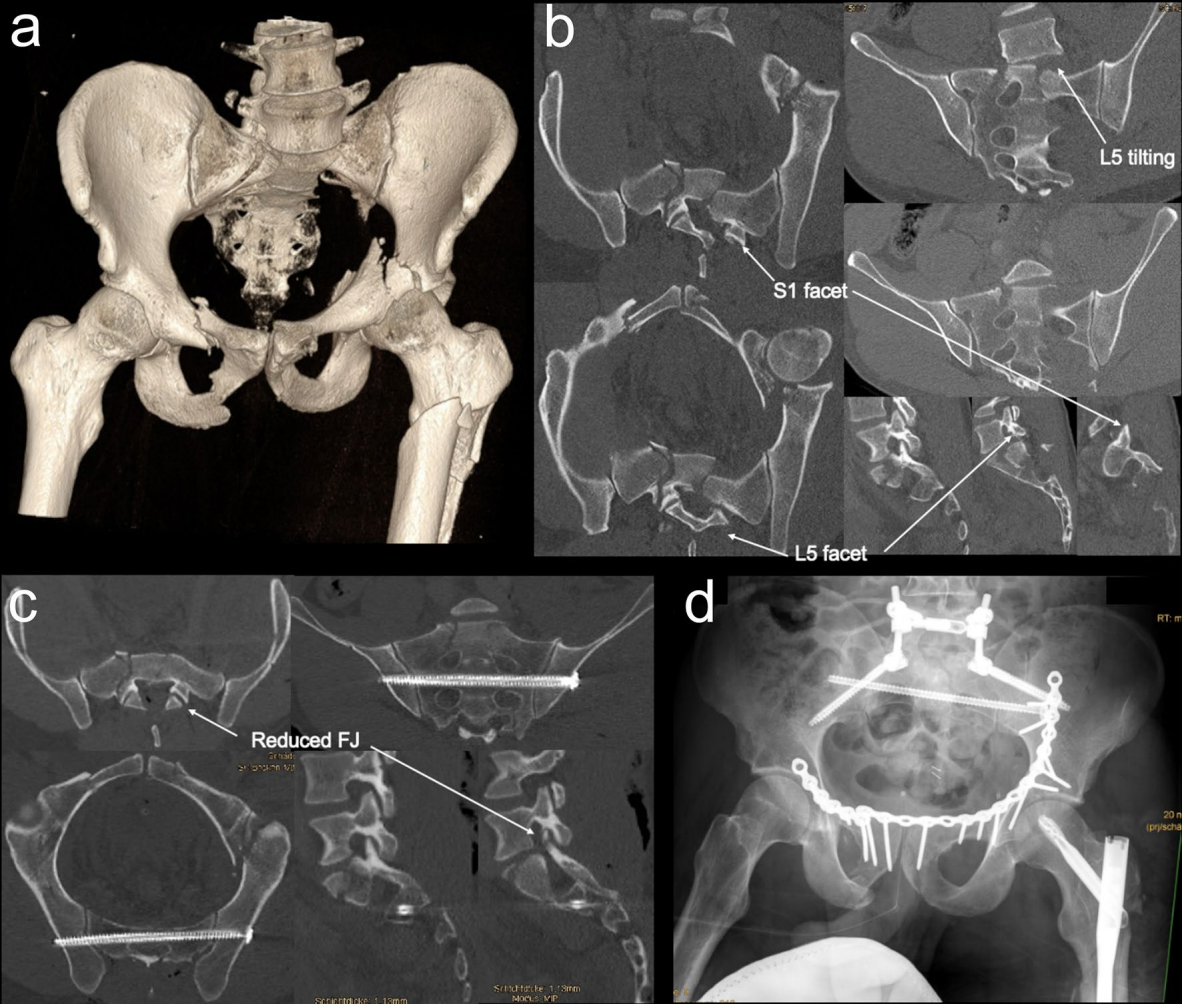
Bilateral vertical sacral fracture without a transverse fracture component (BVSF; Type 4 TLSI). A 37-year-old male polytrauma patient fell from a height of 8 m in a paragliding accident and presented with left-sided L5 and S1 nerve root dysfunction. **a** 3D CT reconstruction showing a BVSF associated with an anterior pelvic ring injury and a left-sided acetabular fracture. **b** Multiplanar CT reconstructions demonstrating left-sided lumbosacral facet joint dislocation and coronal tilting of the L5 vertebral body. **c** CT images obtained after immediate open reduction, decompression, and dural repair via a posterior midline approach, followed by transsacral screw fixation in S2. **d** AP radiograph after staged anterior pelvic ring and acetabular fracture fixation, as well as additional L5-ilium instrumentation and fusion performed on the following day

In general, pelvic ring fractures with complete unilateral disruption of the posterior arch through the sacrum (AO/OTA 61-C1.3) are stabilized by a combination of anterior and posterior pelvic ring fixation [48]. Regarding the *surgical sequence*, it is generally advisable to start anteriorly if there is a displaced anterior pelvic ring or an associated displaced acetabular fracture requiring reduction and fixation. However, in cases of a dislocated or locked ipsilateral L5/S1 facet joint, or an otherwise irreducible posterior pelvic ring, the sequence should begin posteriorly (Fig. 4).

Various *posterior pelvic ring fixation techniques* have been described to stabilize UVSF, including iliosacral screws, transsacral screws [103], transsacral bars [120], transiliac plates [121, 122], transiliacal internal fixators [123, 124], and direct plate osteosynthesis of the sacrum [125, 126]. All these techniques share the common feature of being applied within the posterior pelvic ring, with implants typically oriented perpendicular to the vertical sacral fracture. Consequently, their ability to resist vertical shear forces is limited, usually necessitating restricted postoperative weight-bearing [86]. Percutaneous screw fixation is the most commonly used technique to stabilize UVSF and allows for minimal soft tissue trauma, negligible blood loss, relatively short operative time, and a low rate of neurovascular complications [103, 127–129]. However, iliosacral screw fixation does not always provide reliable stabilization, and fixation failure is not uncommon, particularly in vertically unstable, comminuted transforaminal UVSF [130–132]. Moreover, while percutaneous screw fixation can restore posterior pelvic ring stability, it does not address lumbosacral instability in UVSF with facet joint disruption.

In contrast to the previously mentioned posterior pelvic ring fixation techniques, *unilateral triangular lumbopelvic fixation (uTLPF)*, also known as triangular osteosynthesis, spans the L5/S1 motion segment and consists of two key components: a vertically oriented L5 to ilium instrumentation and an ipsilateral horizontally oriented iliosacral screw fixation [86]. The former primarily counteracts cephalad migration of the injured, while the latter prevents fracture splaying in the frontal plane and enhances multiplanar rotational stability. Compared to sacral screw fixation, the uTLPF construct provides superior biomechanical stability and allows for earlier full weight-bearing [85]. Most importantly, it enables restoration of both posterior pelvic ring stability and lumbosacral stability in UVSF with ipsilateral L5/S1 facet joint disruption. In high-energy UVSF, there are two primary indications for uTLPF: UVSF with L5/S1 facet joint disruption resulting in TLSI, and highly comminuted transforaminal UVSF, which are at higher risk of fixation failure if only sacral screws are applied [41, 42]. Details of the operative technique are beyond the scope of this article and have been described with some variations by others [41, 42, 86, 133, 134]. Briefly, the fracture is accessed via a posterior midline approach if sacral canal decompression is required, or via a paraspinal Wiltse approach if only foraminal or no decompression is necessary. After the placement of the L5 pedicle screw, decompression is performed as needed, and sacral nerve root entrapment must be evaluated and ruled out prior to any compression maneuvers. Subsequently, the sacral fracture and ipsilateral facet joint are reduced. In our practice, reduction is best achieved via manipulation of a Schanz Pin placed into the ilium of the injured hemipelvis and via pointed reduction clamps. Now, an iliosacral or transsacral screw is placed to secure the reduction. The use of a transsacral screw instead of an iliosacral screw potentially enhances the stability of the uTLPF construct, as indicated by a recent finite element analysis study [135]. The iliac screw is then inserted using the anatomic technique to avoid iliac screw head prominence [136], which, along with soft tissue complications, is among the most common issues following uTLPF [90, 137, 138]. Finally, the uTLPF is completed by attaching the rod to the L5 and iliac screw. In cases of UVSF with facet joint dislocation or residual facet joint incongruence after reduction, the ipsilateral facet joint is fused using autologous or homologous bone grafts. In our experience, the following technical aspects are particularly important: First, we do not use the L5 to ilium instrumentation for reduction, as distraction along the longitudinal connecting rod may lead to overdistraction and tilting of the L5 vertebra in the coronal plane, especially in cases with ipsilateral facet joint involvement [137]. Instead, the L5 to ilium instrumentation is applied as a neutralizing construct after reduction has been secured by the iliosacral screw. Second, symptomatic posterior prominence of the iliac screw head must be avoided through proper iliac screw positioning. Third, placing the iliosacral screw first ensures that the iliac screw does not interfere with the iliosacral screw corridor. In addition to open techniques, less invasive and percutaneous uTLPF approaches have been described [44, 118, 139]. Percutaneous uTLPF may be particularly useful when open reduction or decompression is not required.

Overall, a *specific treatment concept* for UVSF with ipsilateral L5/S1 facet joint involvement has not yet been established. In our view, the treatment strategy should be tailored to the specific pattern of ipsilateral L5/S1 facet joint involvement and its impact on lumbosacral stability. Additionally, other factors—such as the degree of sacral fracture comminution, the pattern of anterior pelvic ring injury, soft tissue status, and the expected time to mobilization—should also be considered. In general, open reduction and uTLPF are particularly indicated for UVSF with ipsilateral L5/S1 facet joint involvement associated with: (1) facet joint dislocation, (2) facet joint subluxation, (3) horizontal S1 facet fracture or L5 inferior articular process fracture, or loose S1 facet (PFL medial and lateral to the S1 facet), (4) significant UVSF comminution or vertical displacement, or (5) neurological deficits due to sacral canal compromise and/or intraforaminal bone fragments requiring neurological decompression. Traditional posterior pelvic ring fixation techniques, such as percutaneous iliosacral or transsacral screw fixation, may be the method of choice for noncomminuted, nondisplaced UVSF with a simple PFL running medial to (or through) the S1 facet and with preserved L5/S1 facet joint congruency without subluxation. As noted by Sagi [42], UVSF with the PFL passing medial to the ipsilateral S1 facet, while the L5/S1 facet joint remains otherwise intact, may exhibit less vertical instability and a lower tendency for further vertical displacement than UVSF with the PFL passing lateral to the ipsilateral S1 facet. In this scenario the ipsilateral S1 facet remains attached to the sacrum lateral to the UVSF, with the intact ipsilateral L5/S1 facet joint providing resistance to vertical displacement of the injured hemipelvis. The role of nonoperative treatment requires further clarification. According to Kanna et al. [13], non- and minimally displaced fractures without comminution and with a stable anterior pelvic ring may even be amenable to nonoperative management. Aside from defining the precise indications for each treatment strategy, other important questions still need to be resolved, including: What is the impact of different ipsilateral facet joint involvement patterns on posttraumatic facet joint osteoarthritis? How does posttraumatic facet joint osteoarthritis contribute to chronic low back pain after UVSF? Which patterns of ipsilateral facet joint involvement necessitate facet joint fusion? Should fusion be performed unilaterally or rather bilaterally? Is anatomic reduction of facet joint dislocation or subluxation without fusion, followed by later removal of uTLPF, a reasonable option to restore lumbosacral stability and regain mobility in the L5/S1 motion segment? What is the optimal postoperative weight-bearing regime for each treatment strategy? When is implant removal indicated?

#### Expected outcomes and complications

To our knowledge, only two retrospective studies have specifically reported outcomes for UVSF with ipsilateral facet joint involvement. Liu et al. [118] analyzed 28 patients, of whom 11 patients underwent robot-assisted percutaneous uTLPF and 17 underwent conventional open uTLPF via a posterior midline approach. Fusion of the injured L5/S1 facet joint was performed in 3 patients in the percutaneous group and 6 patients in the open group due to the inability to achieve anatomical reduction. At a minimum of 12 months post-surgery, the mean Majeed score was 90.7 ± 5.6 in the robot-assisted group and 85.5 ± 5.6 in the conventional group. Three patients (one in the robot-assisted group and two in the conventional group) had to change their physically demanding jobs due to persistent lumbosacral pain. Maximum residual displacement was excellent (0–5 mm) in 82% of patients in both groups. Complications included painful prominent iliac implants (three cases), one deep wound infection, and one deep wound hematoma, both occurring in the conventional group. Kanna et al. [13] retrospectively compared operative versus nonoperative treatment of 34 patients with UVSF and associated ipsilateral lumbosacral facet joint involvement, including 3 Type 1, 13 Type 2a, 15 Type 2b and 3 Type 2c injuries according to Isler’s classification. All patients had concomitant anterior pelvic ring injuries, which were stabilized in 6 cases in the operative group and in 2 cases in the nonoperative group. According to the authors, the decision for operative treatment was based on hemi-pelvis displacement > 1 cm, neurological deficit and extent of anterior pelvic ring injury, but was not further specified. In the operative group, one or two iliosacral screws were used for UVSF with minimal comminution (n = 11), while lumbopelvic fixation (n = 5) or uTLPF (n = 2) was used for severely displaced or severely comminuted UVSF, and for cases necessitating open reduction. At a mean follow-up of 15.2 months (range 6–48 months), all fractures had healed radiographically, and functional outcomes – such as mean VAS score for low back pain, ability to squat and sit cross-legged, return to work, and Majeed score (77.2 ± 3.9 vs 79.6 ± 4.1) – did not significantly differ between the operative (n = 18) and nonoperative (n = 16) groups. Accuracy of reduction was not reported. Complications were limited to nonsurgical issues, such as urinary tract infections. Other studies have reported outcomes of open or percutaneous uTLPF but have either not specifically addressed or only anecdotally mentioned ipsilateral lumbosacral facet joint involvement [44, 86, 90, 133, 134, 137, 139, 140].

### Surgical management of bilateral vertical sacral fractures without a transverse fracture component (BVSF; Type 4 TLSI)

Bilateral complete vertical sacral fractures without a transverse fracture component (BVSF) represent the fourth type of TLSI. These fractures result in both PPRI and TLSI because the central portion of the sacrum (medial to both UVSF) remains connected to the spine but is mechanically disconnected from both hemipelves. This injury pattern corresponds to a C3.3 injury according to the AO/OTA 2018 pelvic ring classification and a subtype C2 injury according to the AOSpine Sacral Classification System. BVSF are often confused with SPDI, particularly the H-variant, because the transverse fracture component is underappreciated and insufficiently recognized. However, a study by Bishop et al. [141] found that 87% of bilateral vertical sacral fractures involve a transverse fracture component, classifiying them as SPDI rather than BVSF. Since this study also included elderly patients, it is reasonable to assume that the percentage of bilateral vertical sacral fractures without a transverse fracture component would have been even lower if only high-energy injuries in younger patients had been considered. Additionally, the study highlighted the rarity of BVSF, accounting for only 1% (6/490) of all uni- or bilateral vertical sacral fractures.

Despite the general consensus that surgical fixation is indicated in BVSF, the scientific literature on the optimal treatment for this rare injury pattern remains extremely limited, and no standardized treatment protocol has been established. Furthermore, the inclusion of C3.3 injuries is only sporadically specified in studies on the treatment of unstable posterior pelvic ring disruptions, and it is often not explicitly stated whether a transverse fracture component was excluded (see below).

The *aims* of operative treatment of BVSF include realignment of the bony architecture in cases of significant displacement, restoration of posterior pelvic ring and lumbosacral stability, facilitation of early mobilization, and neurologic decompression in cases of neurologic deficits due to sacral canal or neuroforaminal encroachment.

In general, various potential *treatment options* exist for BVSF, including percutaneous screw fixation, bilateral triangular lumbopelvic fixation (TLPF) or lumbopelvic fixation (LPF), and others, such as transiliac plating. As mentioned above, iliosacral *screw fixation* does not always provide reliable stabilization in type C UVSF, particularly in cases with comminution or residual fracture gaps. Biomechanical studies have demonstrated that using two iliosacral screws in vertically unstable UVSF provides greater rotational stiffness and load to failure compared to a single screw [142, 143]. Transsacral screws, also referred to as transiliac-transsacral screws, may provide enhanced fixation due their greater length, improved load distribution, and ability to engage multiple cortices [84, 103, 144–147]. This technique has been proposed as an effective method for posterior pelvic ring fixation, even in cases of bilateral posterior pelvic injuries [84, 103, 148]. Limited clinical and biomechanical data suggests that the use of two transsacral screws offers more reliable fixation than a single screw, with fully threaded screws providing superior biomechanical stability compared to partially threaded ones [87, 149, 150]. Consistent with these findings, finite element analysis studies on BVSF indicate that, in terms of stability and risk of screw breakage, percutaneous screw fixation of both the S1 and S2 segment is superior to fixation of either S1 or S2 alone, and the use of transsacral screws for S1 and S2 fixation is biomechanically superior to bilateral iliosacral screws [151, 152]. Consequently, transsacral screw fixation, involving at least one transsacral screw in S1 and S2, may be a viable option for the fixation of non- or minimally displaced BVSF without significant comminution, lumbosacral facet joint disruption and neural encroachment. However, percutaneous screw placement may be exceedingly difficult or precluded by narrowed or absent safe zones due to imperfect fracture reduction, while sacral dysmorphism may restrict transsacral screw placement to S2, requiring the use of bilateral iliosacral screws in S1. Furthermore, percutaneous screw fixation is oriented perpendicular to the weight-bearing axis, limiting its ability to resist vertical shear forces. As a result, postoperative weight-bearing is typically restricted for a considerable period following sacral screw fixation. It remains unclear whether transsacral screw fixation in BVSF permits early full weight-bearing, which is ultimately inevitable. In unilateral vertically unstable sacral fractures, partial weight-bearing is possible because the contralateral weight-bearing axis remains intact. However, in BVSF, the disruption of both weight-bearing axes renders partial weight-bearing impossible. In BVSF, the axial load of the upper body is transferred from the lumbar spine to the central sacrum, leading to caudal migration of the central sacrum relative to both hemipelves and generating a flexion moment of the central sacrum (rotation in the sagittal plane). Therefore, transiliac plating, which does not involve fixation of the central sacrum, appears ineffective for stabilizing this multidirectional instability, although its use has been reported sporadically in patients with C3.3 injuries [121, 153, 154].

From a biomechanical perspective, bilateral triangular lumbopelvic fixation (*TLPF*) and lumbopelvic fixation (*LPF*) provide the greatest biomechanical stability for sacral fracture fixation and enable early full weight-bearing [85, 87, 155, 156]. Fixation is achieved cranially through bilateral L5 pedicle screws and caudally with long screws in the ilium. This construct spans the sacrum and restores the integrity of load transfer from the lumbar spine to the iliac bones, bypassing the sacrum and sacro-iliac joints. In TLPF, the vertically oriented bilateral L5 to ilium instrumentation primarily resist caudal migration of the central sacrum relative to both hemipelves, whereas the horizontally oriented transsacral (or iliosacral) screws predominantly counteract fracture splaying and flexion of the central sacrum in the sagittal plane. In LPF, the horizontally oriented fixation component consists of a transverse connector rather than a sacral screw. These two stabilization techniques provide the most reliable fixation for BVSF and are particularly indicated in cases with significant comminution, uni- or bilateral lumbosacral facet joint disruption, or significant displacement or neural encroachment requiring open reduction and decompression (Fig. 4). Percutaneous TLPF can be employed in cases where open reduction or decompression is not required, reducing the rate of soft tissue complications associated with open techniques [81, 107, 157]. Even in non- or minimally displaced BVSF, (percutaneous) TLPF may be considered the preferred treatment, particularly when associated injuries allow for early patient mobilization.

#### Expected outcomes and complications

Few studies on the treatment of unstable posterior pelvic ring disruptions explicitly mentioned that some C3.3 injuries according to AO/OTA (or the more vaguely termed “bilateral sacral fractures”) were included. However, these studies typically reported combined outcomes of a broader group of injuries, rather than focusing specifically on C3.3 injuries, and often did not clarify whether presence of a transverse fracture component was excluded [97, 103, 121, 133, 148, 153, 154, 158–160]. To the best of our knowledge, only one retrospective study specifically addressed the treatment of bilateral sacral fractures: Wenning et al. [161] reported on 77 patients (20 males, 57 females) with unstable bilateral sacral fractures, of whom 29 underwent LPF and 48 underwent bilateral iliosacral screw (IS) fixation in combination with uni- or bilateral superior pubic ramus screw fixation. Patients in the IS group were older than those in the LPF group (75.9 ± 14.0 vs. 62.2 ± 17.7 years), while body mass index and comorbidities were comparable. The fracture patterns included 37 C0 (48%; nondisplaced SPDI), 26 C2 (34%; BVSF), and 14 C3 (18%; displaced SPDI) injuries according to the AOSpine Sacral Classification System. The mechanism of injury (high- vs. low-energy) was not specified. The authors found that operative time and length of hospital stay was shorter in the IS group, but the mean time to start weight-bearing was longer compared to the LPF group (7.0 vs. 0.83 weeks, respectively). The overall complication rate did not differ between groups; however, the rate of operative debridement due to deep wound infection or hematoma was higher in the LPF group (4 patients (13.8%) vs. 1 patient (2.1%), respectively). It is important to note that the mean age of patients was relatively high (71 years, range 19 to 97 years), likely indicating that a substantial portion of cases involved low-energy fragility fractures of the pelvis. Additionally, all 26 BVSF (subtype C2) were treated by bilateral iliosacral screws, while LPF was only applied in SPDI cases, whether nondisplaced or displaced.

## Authors’ preferred treatment approach

Although current evidence is insufficient to support definitive, evidence-based treatment recommendations, we propose a simplified treatment algorithm for these rare injuries associated with TLSI. Grounded in available data and clinical experience, this approach offers a practical framework to assist in decision-making until more robust evidence becomes available. Nevertheless, patient-specific factors, such as physiological status, lumbosacral and gluteal soft tissue conditions, and expected time to mobilization, may require individualized modifications to this approach.

### Lumbosacral dislocation injuries (LSDI; Type 1 TLSI)

- *LSDI with low-grade slip and intact L5/S1 disc* (as verified by preoperative MRI; e.g., unilateral lumbosacral pure facet dislocation)
  Preferred treatment**:** Open decompression, reduction, posterior L5-S1 instrumentation and posterior/posterolateral fusion
  - Decompression may be omitted in patients without neurological deficits
  - Circumferential fusion, including interbody fusion (IF), may be considered even in the presence of an intact disc if decompression involves substantial posterior bone resection
  - Posterolateral fusion might be compromised by fractured and displaced L5 transverse processes, frequently encountered in LSDI
- *LSDI with L5/S1 disc injury* (as verified by preoperative MRI or indicated by high-grade slip)
  Preferred treatment**:** Open decompression, reduction, posterior L5-S1 instrumentation and circumferential fusion, including interbody fusion (IF)
  - IF is typically performed via the posterior approach; however, anterior IF may be considered, particularly in severely unstable cases, such as traumatic lumbosacral spondyloptosis
  - Extension of posterior instrumentation to L4 and ilium may be considered in severely unstable cases, such as traumatic lumbosacral spondyloptosis

### Spinopelvic dissociation injuries (SPDI; Type 2 TLSI)

- *Minimally displaced Type 1 and Type 2 SPDI*
  Preferred treatment**:** Percutaneous in situ fixation using percutaneous triangular lumbopelvic fixation (TLPF) or transsacral screw fixation
  - Transsacral screw fixation is particularly considered for U-shaped coronal fracture patterns with suitable osseous corridors allowing placement of at least two transsacral screws cranial to the transverse fracture component
  - Percutaneous TLPF is preferred in H-, Y- and lambda-shaped patterns, as well as U-shaped patterns lacking adequate osseous fixation pathways for transsacral screws. In the latter scenario, bilateral iliosacral screws are used instead of a transsacral screw.
  - “Minimal displacement” is poorly defined but may include kyphotic angulation of less than 10°–20°. In general, restoration of physiological alignment of the upper central sacrum is the goal, and thus a low threshold is applied when indicating closed reduction.
- *Significantly displaced U-shaped Type 1 and Type 2 SPDI without* *significant comminution and neural encroachment by free bone fragments in the sacral canal or neuroforamina*
  Preferred treatment**:** Closed reduction and percutaneous TLPF
  - H-, Y-, and lambda-shaped patterns are considered for closed reduction only if their vertical fracture components are not comminuted, unstable, or displaced
  - If adequate reduction cannot be achieved, closed reduction attempts are followed by open reduction
- *Significantly displaced Type 1 and Type 2 SPDI with any of the following***: **(1) unstable or displaced vertical fracture components (in H-, Y-, and lambda-shaped patterns); (2) comminuted transverse or vertical fracture components with neural encroachment caused by loose bone fragments within the spinal canal or neuroforamina; (3) significant residual displacement or persistent neural impingement after closed reduction attempts; (4) inadequate kyphosis correction through indirect methods, requiring direct manipulation of the upper central sacrum; (5) associated L5/S1 facet joint disruption requiring fusion
  Preferred treatment**:** Open reduction ± direct decompression, and bilateral TLPF or LPF
- *Type 3 SPDI*
  Preferred treatment**:** Open reduction ± direct decompression, and bilateral TLPF or LPF
  - Percutaneous in situ fixation may be considered for selected cases

### Unilateral vertical sacral fractures with ipsilateral L5/S1 facet joint involvement

- *Noncomminuted, nondisplaced UVSF with a simple PFL running medial to (or through) the S1 facet and with preserved L5/S1 facet joint congruency without subluxation*
  Preferred treatment**:** Percutaneous iliosacral or transsacral screw fixation
  - Nonoperative treatment, as suggested by Kanna et al. [13], may be considered for selected cases
- *UVSF with ipsilateral L5/S1 facet joint involvement and any of the following***: **(1) facet joint dislocation; (2) facet joint subluxation; (3) horizontal S1 facet fracture or L5 inferior articular process fracture, or loose S1 facet (PFL medial and lateral to the S1 facet); (4) significant UVSF comminution and vertical displacement; (5) neural encroachment
  Preferred treatment**:** Open reduction and unilateral TLPF (uTLPF) (including ipsilateral lumbosacral facet joint fusion in cases of facet joint disruption and decompression in cases of neural encroachment)
  - Percutaneous uTLPF may be considered in cases with significant UVSF comminution but preserved facet joint congruency

### Bilateral vertical sacral fractures without a transverse fracture component (BVSF)

- *Non- or minimally displaced BVSF without lumbosacral facet joint disruption or neural encroachment*
  Preferred treatment: Percutaneous TLPF
  - Particularly recommended in cases with significant comminution or when associated injuries permit early full weight-bearing
  - Percutaneous screw fixation alone with at least two transsacral screws (preferably in S1 and S2; or at least one transsacral screw in S2 and bilateral iliosacral screws in S1 in cases of sacral dysmorphism) may be considered in cases without significant comminution
- *Displaced or comminuted BVSF requiring open reduction and/or decompression, or BVSF with associated lumbosacral facet joint disruption*
  Preferred treatment**:** Open reduction, and bilateral TLPF or LPF (including lumbosacral facet joint fusion in cases of facet joint disruption and decompression in cases of neural encroachment)

Concomitant anterior pelvic ring injuries are common in Type 2 to 4 TLSI. The indication for anterior pelvic ring stabilization (e.g., plate fixation, retrograde superior pubic ramus screw fixation, or anterior external/internal fixators) depends on the specific pelvic ring injury type. As these injuries commonly correspond to type C pelvic ring injuries according to the AO/OTA 2018 classification, anterior pelvic ring stabilization is often indicated to (1) enhance overall stability and reduce the risk of posterior implant failure, and (2) prevent secondary displacement of the anterior pelvic ring that may lead to facet joint incongruency in Type 3 TLSI. In general, it is advisable to begin with anterior stabilization if there is an associated displaced anterior pelvic ring or acetabular fracture requiring reduction and fixation. Exceptions include cases with locked ipsilateral L5/S1 facet joints or an otherwise irreducible posterior pelvic ring, as outlined above.

Regarding postoperative weight-bearing protocols, patients are generally permitted immediate weight-bearing as tolerated and as allowed by any associated lower extremity injuries.

## Conclusion

The four complex injury patterns underlying TLSI continue to pose significant challenges for both pelvic and spine surgeons. In this article, we outlined the key factors that influence decision-making in the early clinical phase. We systematically reviewed the currently available treatment options, aiming to provide a structured framework for guiding therapeutic decisions. Selecting the most appropriate treatment strategy for each patient requires careful consideration of both the specific injury characteristics and relevant clinical factors, including the patient’s physiological and neurological status, soft-tissue condition, and anticipated time to mobility.

## Data Availability

No datasets were generated or analysed during the current study.
